# Cerebroprotective Effect of 17β-Estradiol Replacement Therapy in Ovariectomy-Induced Post-Menopausal Rats Subjected to Ischemic Stroke: Role of MAPK/ERK1/2 Pathway and PI3K-Independent Akt Activation

**DOI:** 10.3390/ijms241814303

**Published:** 2023-09-19

**Authors:** María C. Burguete, Teresa Jover-Mengual, María Castelló-Ruiz, Mikahela A. López-Morales, José M. Centeno, Alicia Aliena-Valero, Enrique Alborch, Germán Torregrosa, Juan B. Salom

**Affiliations:** 1 Unidad Mixta de Investigación Cerebrovascular, Instituto de Investigación Sanitaria La Fe, Universitat de València, 46100 Burjassot, Spain; m.consuelo.burguete@uv.es (M.C.B.); maria.castello@uv.es (M.C.-R.); mikahela_lopez@iislafe.es (M.A.L.-M.); jose.m.centeno@uv.es (J.M.C.); alicia_aliena@iislafe.es (A.A.-V.); enrique.alborch@uv.es (E.A.); torregrosa_ger@gva.es (G.T.); salom_jba@gva.es (J.B.S.); 2 Departamento de Fisiología, Universitat de València, 46100 Burjassot, Spain; 3 Departamento de Biología Celular, Biología Funcional y Antropología Física, Universitat de València, 46100 Burjassot, Spain; 4 Hospital Universitari i Politècnic La Fe, 46026 Valencia, Spain

**Keywords:** 17β-estradiol, MAPK/ERK, PI3K/Akt, neuronal death, apoptosis, ischemic stroke, neuroprotection

## Abstract

Despite the overwhelming advances in the understanding of the pathogenesis of stroke, a devastating disease affecting millions of people worldwide, currently there are only a limited number of effective treatments available. Preclinical and clinical studies show that stroke is a sexually dimorphic disorder, affecting males and females differently. Strong experimental evidence indicates that estrogen may play a role in this difference and that exogenous 17β-estradiol (E2) is neuroprotective against stroke in both male and female rodents. However, the molecular mechanisms by which E2 intervenes in ischemia-induced cell death, revealing these sex differences, remain unclear. The present study was aimed to determine, in female rats, the molecular mechanisms of two well-known pro-survival signaling pathways, MAPK/ERK1/2 and PI3K/Akt, that mediate E2 neuroprotection in response to acute ischemic stroke. E2 pretreatment reduced brain damage and attenuated apoptotic cell death in ovariectomized female rats after an ischemic insult. Moreover, E2 decreased phosphorylation of ERK1/2 and prevented ischemia/reperfusion-induced dephosphorylation of both Akt and the pro-apoptotic protein, BAD. However, MAPK/ERK1/2 inhibitor PD98059, but not the PI3K inhibitor LY294002, attenuated E2 neuroprotection. Thus, these results suggested that E2 pretreatment in ovariectomized female rats modulates MAPK/ERK1/2 and activates Akt independently of PI3K to promote cerebroprotection in ischemic stroke. A better understanding of the mechanisms and the influence of E2 in the female sex paves the way for the design of future successful hormone replacement therapies.

## 1. Introduction

Ischemic stroke remains as a major disease, representing a huge medical, social, and economic burden worldwide [1]. As it is widely documented that stroke is a sex-dimorphic disease, emphasis is placed on the need for a better understanding of the mechanisms behind the differences in stroke pathophysiology, as well as on how current treatment options affect both sexes to improve stroke care in women [2,3,4]. Sex differences are reported to occur in the epidemiology, risk, and response to stroke [5,6,7]. In fact, stroke in women may be considered as a distinct entity due to numerous differences compared with men, and health policies need to be adapted accordingly to improve stroke prevention and pre-stroke health in women [4]. Furthermore, experimental findings show that male and female cells do not react the same way to death or survival messages after insult [8,9]. In addition, the presence or deprivation of estrogen suppresses or magnifies gender differences in physiology and pathophysiology [10,11,12]. Finally, we and others have reported that both sex steroids and gender differences are important components in experimental and clinical strokes that can affect the mechanisms and outcomes of cerebral ischemia [13,14].

Neurosteroids are a kind of steroids synthetized in neurons and glia, with relevant functions in the neural tissue under both physiological and pathological conditions. Since Simpkins and colleagues (1997) provided the first evidence that 17β-estradiol (E2) exerted neuroprotective effects in the transient middle cerebral artery occlusion (tMCAO) model [15], its neuroprotective action has been repeatedly demonstrated in a variety of experimental models of brain damage including ischemic stroke [16,17]. We have previously shown that acute E2 treatment significantly lowers total infarct volume in the tMCAO model in male rats [18] by inhibiting apoptosis, an effect associated with an increase in the expression of the estrogen receptor (ERα) but not with the activation of either the MAPK/ERK1/2 or the PI3K/Akt pathways [19]. By contrast, later, we demonstrated that E2 attenuated the ischemia-induced increase in ERK1/2 activity under diabetes condition in male rats [20].

E2 exerts its effect through the activation of three types of estrogen receptors (ER): ERα and ERβ, both localized in the intracellular compartment as well as membrane-associated receptors, and G protein-coupled estrogen receptor 1 (GPER1), described as a transmembrane receptor. Estrogenic signaling can follow a “classical” (slow, transcription-dependent) and a “non-classical” (fast, cytoplasmic-dependent) pathway. Rapid estrogenic action is associated with growth factor signaling such as the mitogen-activated protein kinase (MAPK) and phosphoinositide 3-kinase (PI3K) pathways, including their respective immediate downstream effectors, extracellular signal regulated kinases 1 and 2 (ERK1/2), and the serine-threonine protein kinase B (Akt). Both are well-characterized intracellular signaling cascades implicated in the regulation of genes and neuronal apoptosis or survival [21,22,23,24,25]. Interestingly, the current status of cerebroprotection in acute ischemic stroke now includes the emerging concept of “help-me signaling”, which reflects the fact that neurons seek assistance during ischemia by secreting molecules like E2 able to act on adjacent cells [26]. Neurons appear to secrete E2 that activate adjacent astrocytes in a paracrine manner, thus providing neuroprotection and cognitive preservation following ischemic injury to the brain [27].

The current study was designed to reproduce the clinical scenario of menopausal women following a program of hormone replacement therapy (HRT) with E2, then assessing whether such pretreatment confers cerebroprotection against an eventual episode of ischemic stroke. This was performed by using ovariectomized female rats pretreated with E2 replacement and subsequently subjected to tMCAO, a stroke model that mimics the endovascular thrombectomy technique already in use in the clinical setting. Therefore, such an approach refers to a preventive instead of an acute treatment. One could argue that HRT carries significant risks, including risk of stroke, and that there are several nuances to prescribing HRT. However, HRT is considered the gold standard for management of vasomotor and gynecological symptoms of menopause, and a variety of variables (cyclic/continuous administration, tapering therapy, dosage forms, combination with other estrogens/estrogen receptor modulators, etc.) should be carefully evaluated for optimal treatment in order to minimize HRT-associated risks [28]. We aimed to gain insight into the molecular mechanisms of E2-elicited cerebroprotection so that it could help, along with the above-mentioned variables, in the decision-making process of HRT treatment. This study simultaneously compares the patterns of activation of the MAPK/ERK1/2 and PI3K/Akt signaling pathways, which can mediate E2 neuroprotection in cerebral ischemia, in ovariectomy-induced post-menopausal female rats.

## 2. Results

### 2.1. Estradiol Afforded Neuroprotection against Focal Ischemia-Induced Cerebral Infarction in Ovariectomized Female Rats

**Figure 1:**
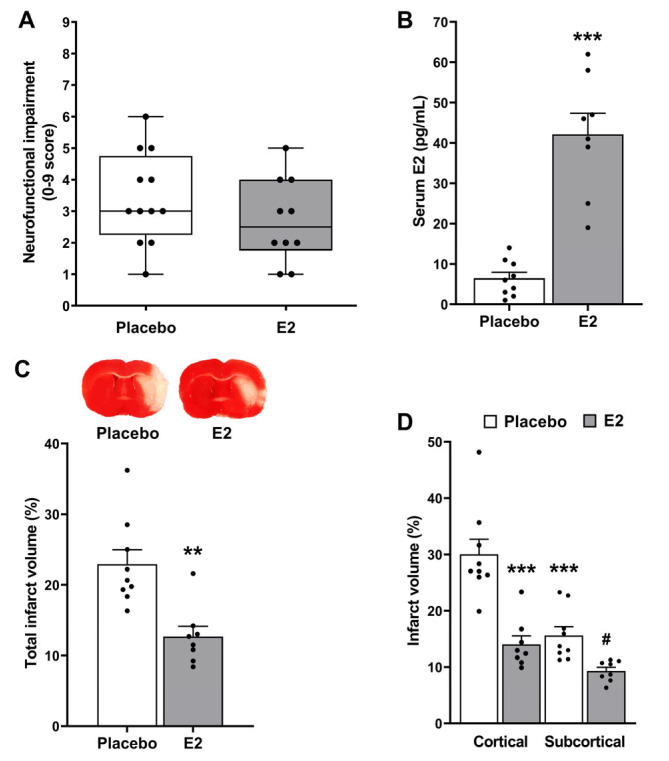
Estradiol decreases infarct volume following tMCAO. (**A**) Effect of E2-treatment on ischemic stroke-induced neurofunctional impairment in ovariectomized female rats subjected to tMCAO (*p* = 0.27, U = 43, Mann–Whitney test). (**B**) Effect of E2-treatment on serum E2 levels. Significantly different from placebo-treated group (*** *p* < 0.001, t = 6.9, DF = 15, Student’s *t*-test). (**C**) Representative images of the TTC-stained third coronal brain slice (0.2/-1.8 mm from Bregma) from placebo- and E2-treated animals, and summary data showing total infarct volumes (expressed as a percentage of the corresponding non-ischemic hemisphere). Significantly different from placebo-treated group (** *p* < 0.01, t = 4, DF = 15, Student’s *t*-test). (**D**) Summary data showing cortical and subcortical infarct volumes. Two-way ANOVA: treatment (E2 vs. placebo, *p* < 0.001, F_1,30_ = 38), brain region (subcortical vs. cortical, *p* < 0.001, F_1,30_ = 28), and interaction (*p* < 0.05, F_1,30_ = 7.1). Post hoc Sidak’s multiple comparisons test: significantly different from cortical infarct volume of placebo-treated animals (*** *p* < 0.001) and significantly different from subcortical infarct volume of placebo-treated animals (^#^ *p* < 0.05). Data are median (Q1, Q3) (box-and-whisker plot) or mean ± SEM (bar graphs) of individual data points. E2, 17β-estradiol. TTC, 2,3,5-triphenyltetrazolium chloride. tMCAO, transient middle cerebral artery occlusion.

At 24 h after the onset of ischemia, neurofunctional examination of fully recovered ovariectomized rats revealed that there was a slight tendency towards improvement of the neurofunctional status of the E2-treated animals (neurological score, 2.5 [1.75, 4]) compared with the placebo group (3 [2.25, 4.75]). However, this improvement was not statistically significant (*p* = 0.27; Figure 1A). Serum estradiol levels at the time of sacrifice were 6.4 ± 1.5 pg/mL in the placebo group and significantly increased in the E2 group (42.1 ± 5.2 pg/mL, *p* < 0.001; Figure 1B).

In the morphometric analysis, animals without TTC-detectable brain infarction were excluded (Table 1). In animals showing clearly delineated pale zones, indicative of macroscopic brain infarction, E2-treated animals showed a significant reduction in the total infarct volume (12.7 ± 1.4% with respect to the volume of the non-ischemic hemisphere) compared to placebo-treated animals (22.9 ± 2.1%, *p* < 0.01). This means an overall reduction in infarct volume of about 50% (Figure 1C). In a regional analysis (Figure 1D), infarct volume was significantly different depending on the treatment (E2 vs. placebo, *p* < 0.001) and the brain region (subcortical vs. cortical, *p* < 0.001), with a significant interaction between both factors (*p* < 0.05). In the post hoc analysis, placebo-treated animals showed significantly smaller infarct volume in the subcortical than in the cortical region (*p* < 0.001). Regarding the treatment, E2-induced reduction in infarct volume was extremely significant in the cortical region (*p* < 0.001) and, to a smaller extent, in the subcortical region (*p* < 0.05).

### 2.2. Apoptosis Inhibition Is a Mechanism of E2-Induced Neuroprotection in Focal Cerebral Ischemia

**Figure 2:**
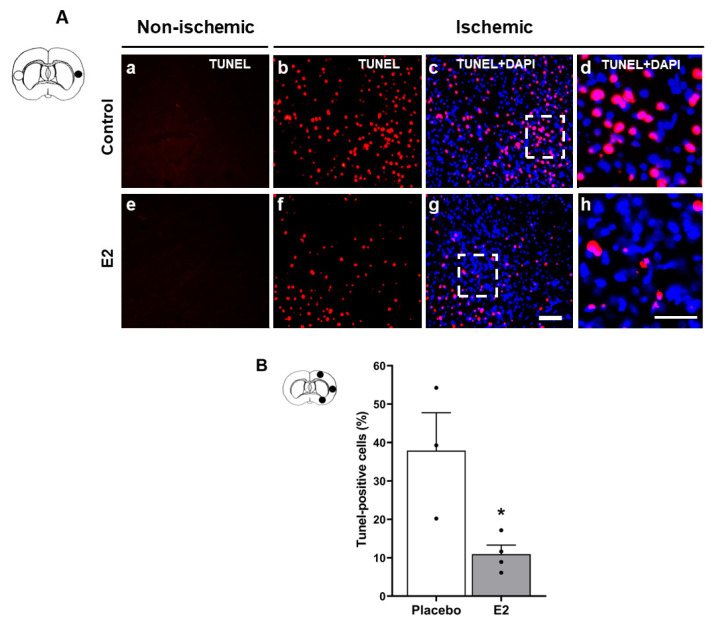
Estradiol attenuates ischemia/reperfusion-induced TUNEL-detected apoptosis. TUNEL detection of apoptotic DNA cleavage in brains from placebo- and E2-treated ovariectomized female rats subjected to tMCAO. (**A**) Lack of TUNEL-labeling (**a**,**e**) in a cortex field of the non-ischemic hemisphere (○), and double-labeling (TUNEL, (**b**,**f**), and TUNEL + DAPI merged images, (**c**,**g**)) in a cortex field of the ischemic hemisphere (●) of representative brain sections. Scale bar, 50 µm. Insets of TUNEL + DAPI merged images (**d**,**h**) are shown at higher magnification. Scale bar, 25 µm. (**B**) TUNEL-positive cell counts expressed as a percentage of the corresponding DAPI-stained nuclei in three selected fields of the ischemic hemisphere (●). Significantly different from placebo group (* *p* < 0.05, t = 3.1, DF = 5, Student’s *t*-test). Data are mean ± SEM of individual data points. E2, 17β-estradiol. tMCAO, transient middle cerebral artery occlusion. TUNEL, terminal deoxynucleotidyl transferase dUTP nick end labeling.

TUNEL-positive cells were undetectable in the cortex of the non-ischemic hemisphere of rats subjected to tMCAO, whether or not they were treated with E2 (Figure 2A(a,e)). By contrast, ischemia/reperfusion induced the presence of TUNEL-positive cells in the ischemic hemisphere cortex of both placebo- (Figure 2A(b–d)) and E2-treated groups (Figure 2A(f–h)). However, ischemic hemisphere from E2-treated animals showed significantly lower TUNEL-positive cell counts (10.9 ± 2.4%) compared with those from placebo-treated animals (37.9 ± 9.9%, *p* < 0.05). Overall, this represented a decrease in approximately 74% (Figure 2B).

**Figure 3:**
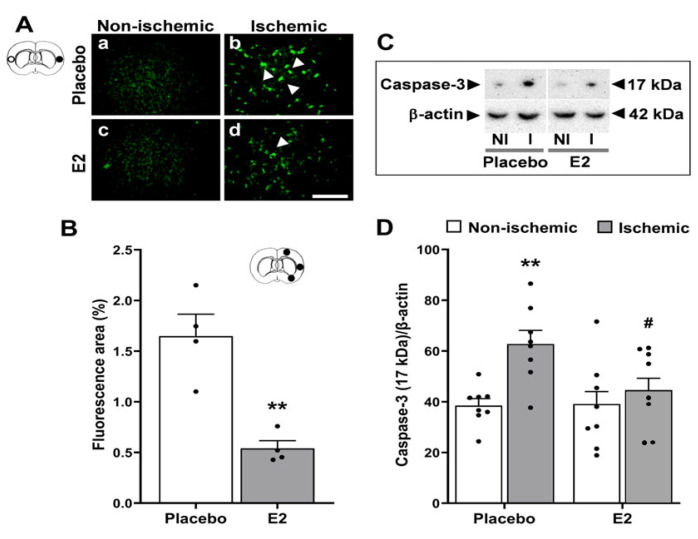
Estradiol inhibits ischemia/reperfusion-induced caspase-3-mediated apoptosis. Apoptosis executor caspase-3 expression in brains from placebo- and E2-treated ovariectomized female rats subjected to tMCAO. (**A**) FAM-DEVD-FMK labeling in a cortex field of the non-ischemic hemisphere (○, (**a**,**c**)) and the ischemic hemisphere (●, (**b**,**d**)) of representative brain sections. Scale bar, 25 µm. (**B**) Fluorescence quantification within three fields of the ischemic cortex (●), expressed as a percentage of the field area. Significantly different from placebo group (** *p* < 0.01, t = 4.8, DF = 6, Student’s *t*-test). Data are mean ± SEM of individual data points. (**C**) Representative Western blots and (**D**) relative abundance of activated caspase-3 p17 form in whole-cell lysates of non-ischemic (NI) and ischemic (I) hemispheres. Two-way ANOVA: ischemic vs. non-ischemic hemisphere *p* < 0.01, F_1,28_ = 9.7. Post hoc Sidak’s multiple comparisons test: significantly different from non-ischemic placebo group (** *p* < 0.01) or from ischemic placebo group (^#^ *p* < 0.05). Data are mean ± SEM of individual data points (normalized to β-actin). E2, 17β-estradiol. tMCAO, transient middle cerebral artery occlusion.

In close agreement with the results from the TUNEL assay, caspase-3 activity was undetectable in the non-ischemic hemisphere (Figure 3A(a,c)). By contrast, ischemia/reperfusion induced caspase-3 activity in the ischemic hemisphere from placebo (Figure 3A(b)) and E2-treated groups (Figure 3A(d)). Once again, marked differences among groups were statistically significant as caspase-3 activity was strongly inhibited in the ischemic hemisphere of E2-treated rats (0.5 ± 0.08%) vs. placebo (1.7 ± 0.2%, *p* < 0.01, Figure 3B). Accordingly, Western blot studies showed significantly higher levels of activated caspase-3 p17 in the ischemic vs. non-ischemic hemisphere (*p* < 0.01). In the post hoc analysis, activated caspase-3 p17 significantly increased in the ischemic hemisphere (62.8 ± 5.3) vs. non-ischemic hemisphere (38.5 ± 2.7, *p* < 0.01) of placebo-treated group. In the ischemic hemisphere, E2 pretreatment significantly reduced activated caspase-3 p17 (45.7 ± 5.5) vs. placebo (62.8 ± 5.3, *p* < 0.05, Figure 3C,D).

### 2.3. E2 Modulated MAPK/ERK1/2 Signaling after Ischemia/Reperfusion

**Figure 4:**
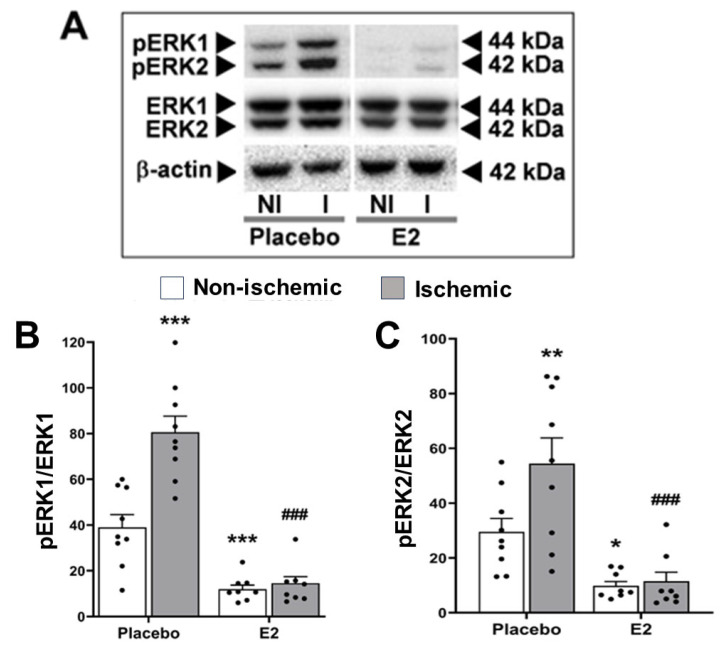
Estradiol decreases phosphorylation of ERK1/2. Phosphorylated extracellular signal-regulated kinase 1/2 (pERK1/2) protein expression in brains from placebo- and E2-treated ovariectomized female rats subjected to tMCAO. (**A**) Representative Western blots and (**B**) relative abundance of pERK1 and (**C**) pERK2 in whole-cell lysates of non-ischemic (NI) and ischemic (I) hemispheres. Two-way ANOVA for pERK1: brain hemisphere (ischemic vs. non-ischemic, *p* < 0.001, F_1,30_ = 18.1), treatment (E2 vs. placebo, *p* < 0.001, F_1,30_ = 84.6), and interaction (*p* < 0.001, F_1,30_ = 15.5). Two-way ANOVA for pERK2: brain hemisphere (ischemic vs. non-ischemic, *p* < 0.05, F_1,30_ = 4.7) and treatment (E2 vs. placebo, *p* < 0.001, F_1,30_ = 28.1). Post hoc Sidak’s multiple comparisons tests: significantly different from non-ischemic placebo group (* *p* < 0.05, ** *p* < 0.01, and *** *p* < 0.001), or from ischemic placebo group (^###^ *p* < 0.001). Data are mean ± SEM of individual data points (normalized to total ERK1/2). E2, 17β-estradiol. tMCAO, transient middle cerebral artery occlusion.

Ischemia/reperfusion significantly increased phosphorylation of both ERK1 (*p* < 0.001) and ERK2 (*p* < 0.05) vs. non-ischemic hemisphere. Post hoc analysis showed that pERK1 from the placebo-treated group significantly increased in the ischemic hemisphere (80.6 ± 7.0) vs. non-ischemic hemisphere (39.0 ± 5.6, *p* < 0.001 Figure 4A,B). Similarly, pERK2 also significantly increased in the ischemic hemisphere (54.4 ± 9.4) vs. non-ischemic hemisphere (29.5 ± 4.9, *p* < 0.01) from the placebo-treated group (Figure 4A,C).

Regarding estrogen pretreatment, E2 significantly decreased phosphorylation of both ERK1 (*p* < 0.001) and ERK2 (*p* < 0.001) vs. placebo-treated group. There were significant interactions between E2 and ischemia-reperfusion effects on changes in both pERK1 (*p* < 0.001) and, to a lower extent, pERK2 (*p* < 0.05). In the post hoc analysis of the non-ischemic hemisphere, E2 significantly reduced both pERK1 (12.2 ± 1.9) and pERK2 (10.1 ± 1.7) vs. placebo-treatment (39.0 ± 5.6, *p* < 0.001, and 29.5 ± 4.8, *p* < 0.05, respectively, Figure 4A–C). In the ischemic hemisphere, E2 also significantly reduced both pERK1 (13.9 ± 3.1) and pERK2 (10.9 ± 3.6) vs. placebo treatment (80.6 ± 7.0, *p* < 0.001, and 54.4 ± 9.4, *p* < 0.001, respectively, Figure 4A–C).

On the other hand, these changes in pERK1/2 levels induced by ischemia-reperfusion and E2 pretreatment were not accompanied by significant changes in total protein levels of ERK1 (ischemic vs. non-ischemic hemisphere, *p =* 0.68, F_1,30_ = 0.17; E2 vs. placebo treatment, *p =* 0.74, F_1,30_ = 0.11,) or ERK2 (ischemic vs. non-ischemic hemisphere, *p =* 0.74, F_1,30_ = 0.11; E2 vs. placebo treatment, *p =* 0.16, F_1,30_ = 2.00). Representative Western blots in Figure 4A.

### 2.4. E2 Attenuated the Ischemia-Induced Akt Signaling Downregulation

**Figure 5:**
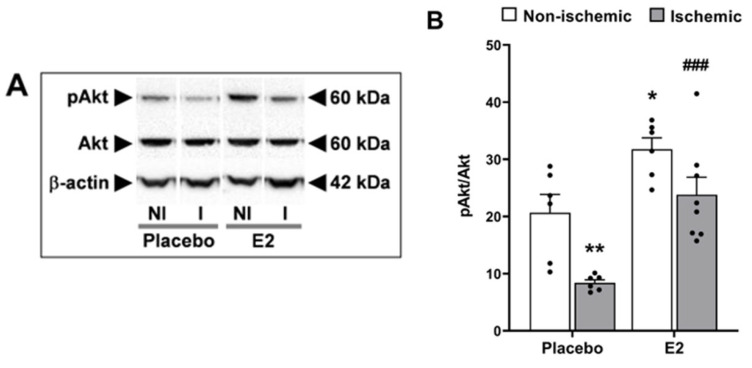
Estradiol blunts the ischemia/reperfusion-induced decrease in Akt phosphorylation. Phosphorylated serine-threonine protein kinase B (pAkt) protein expression in brains from placebo- and E2-treated ovariectomized female rats subjected to tMCAO. (**A**) Representative Western blots, and (**B**) relative abundance of pAkt in whole-cell lysates of non-ischemic (NI) and ischemic (I) hemispheres. Two-way ANOVA: brain hemisphere (ischemic vs. non-ischemic, *p* < 0.001, F_1,22_ = 15) and treatment (E2 vs. placebo, *p* < 0.001, F_1,22_ = 26). Post hoc Sidak’s multiple comparisons test: significantly different from non-ischemic placebo group (* *p* < 0.05 and ** *p* < 0.01), or from ischemic placebo group (^###^ *p* < 0.001). Data are mean ± SEM of individual data points (normalized to total Akt). E2, 17β-estradiol. tMCAO, transient middle cerebral artery occlusion.

We examined the effect of E2 on activation of the pro-survival PI3K/Akt signaling pathway. Ischemia/reperfusion significantly decreased phosphorylation of Akt (pAkt) at Ser473 (*p* < 0.001). Post hoc analysis showed that pAkt from the placebo-treated group significantly decreased in the ischemic hemisphere (8.4 ± 0.6) vs. non-ischemic hemisphere (20.7 ± 3.2, *p* < 0.01, Figure 5A,B). On the other hand, E2 significantly increased phosphorylation of Akt (*p* < 0.001) vs. placebo-treated group. According to the post hoc analysis, E2 significantly increased pAkt when compared to placebo in both the non-ischemic hemisphere (31.8 ± 2.0 in E2 vs. 20.7 ± 3.2 in placebo, *p* < 0.05) and ischemic hemisphere (23.8 ± 3.0 in E2 vs. 8.4 ± 0.6 in placebo, *p* < 0.001, Figure 5A,B), thus attenuating the ischemia-induced decrease in pAkt.

As for total protein levels, Akt expression was not significantly modified by ischemia/reperfusion (*p* = 0.35, F_1,22_ = 0.93) vs. non-ischemic hemisphere. E2 treatment did significantly increase overall expression of total Akt (*p* < 0.01, F_1,22_ = 8.90) vs. placebo treatment. However, the increase was not confirmed by pairwise comparisons in the post hoc analysis (E2 vs. placebo in the non-ischemic hemisphere, *p* = 0.28; E2 vs. placebo in the ischemic hemisphere, *p* = 0.11). Representative Western blots in Figure 5A.

### 2.5. E2 Induced Expression of the Endogenous Akt Inhibitor CTMP in the Non-Ischemic Hemisphere

**Figure 6:**
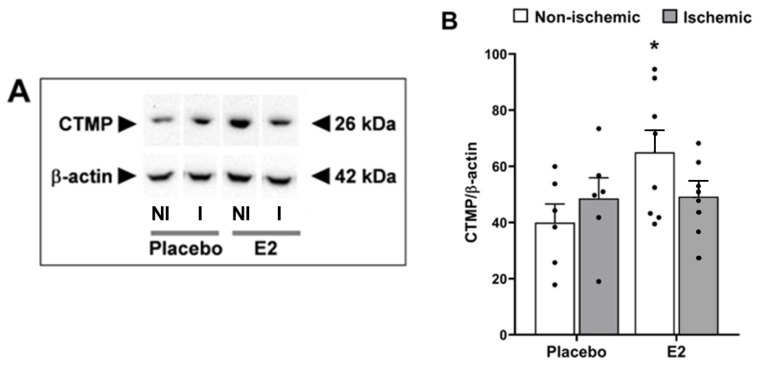
Estradiol increases the CTMP protein expression. Carboxy-terminal modulator protein (CTMP) protein expression in brains from placebo- and E2-treated ovariectomized female rats subjected to tMCAO. (**A**) Representative Western blots and (**B**) relative abundance of CTMP in whole-cell lysates of non-ischemic (NI) and ischemic (I) hemispheres. Two-way ANOVA: E2 vs. placebo, *p* = 0.08, F_1,24_ = 3.1). Post hoc Sidak’s multiple comparisons test: significantly different from non-ischemic placebo group (* *p* < 0.05). Data are mean ± SEM of individual data points (normalized to β-actin). E2, 17β-estradiol. tMCAO, transient middle cerebral artery occlusion.

Both ischemia/reperfusion and E2 treatment have no significant impact on the expression of carboxy-terminal modulator protein (CTMP). However, upon post hoc analysis, it was observed that E2 treatment led to a significant increase in CTMP levels compared to the placebo only in the non-ischemic hemisphere (64.0 ± 8 in E2 vs. 40.0 ± 6.6 in placebo, *p* < 0.05). No notable change was observed in the ischemic hemisphere (48.7 ± 4.6 in E2 vs. 48.6 ± 7.3 in placebo, *p* > 0.99) (Figure 6A,B).

### 2.6. E2 Increased Phosphorylation of the Pro-Apoptotic BAD Protein

**Figure 7:**
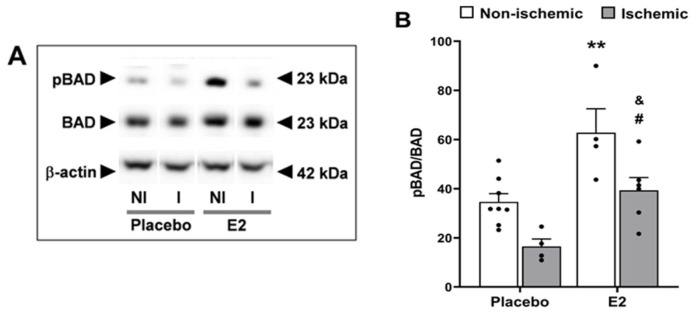
Estradiol increases phosphorylation of the pro-apoptotic protein BAD. Phosphorylated BAD (pBAD) protein expression in brains from placebo- and E2-treated ovariectomized female rats subjected to tMCAO. (**A**) Representative Western blots and (**B**) relative abundance of pBAD in whole-cell lysates of non-ischemic (NI) and ischemic (I) hemispheres. Two-way ANOVA: brain hemisphere (ischemic vs. non-ischemic, *p* < 0.01, F_1,18_ = 15) and treatment (E2 vs. placebo, *p* < 0.001, F_1,18_ = 22). Post hoc Sidak’s multiple comparisons test: significantly different from non-ischemic placebo group (** *p* < 0.01), from ischemic placebo-treated group (^#^ *p* < 0.05), or from non-ischemic E2 group (^&^ *p* < 0.05). Data are mean ± SEM of individual data points (normalized to total BAD). E2, 17β-estradiol. tMCAO, transient middle cerebral artery occlusion.

Ischemia/reperfusion significantly decreased the phosphorylation status of the pro-apoptotic protein BAD at Ser136 (*p* < 0.01) vs. non-ischemic hemisphere. Post hoc analysis showed that pBAD from the E2-treated group significantly decreased in the ischemic hemisphere (39.3 ± 5.2) vs. non-ischemic hemisphere (62.8 ± 9.8, *p* < 0.05, Figure 7A,B). On the other hand, E2 significantly increased phosphorylation of BAD (*p* < 0.001) vs. placebo-treated group. According to the post hoc analysis, E2 significantly increased pBAD when compared to placebo in both the non-ischemic hemisphere (62.8 ± 9.8 in E2 vs. 34.6 ± 3.4 in placebo, *p* < 0.01) and ischemic hemisphere (39.3 ± 5.2 in E2 vs. 16.5 ± 3.0 in placebo, *p* < 0.05, Figure 7A,B), thus attenuating the ischemia-induced decrease in pBAD.

Regarding total protein levels, the changes in pBAD levels induced by ischemia-reperfusion and E2 pretreatment were not accompanied by significant changes in levels of BAD (ischemic vs. non-ischemic hemisphere, *p =* 0.11, F_1,18_ = 2.80; E2 vs. placebo treatment, *p =* 0.40, F_1,18_ = 0.75). Representative Western blots in Figure 7A.

### 2.7. Inhibition of the MAPK/ERK Signaling Pathway, but Not of the PI3K/Akt Signaling Pathway, Attenuated the E2-Induced Cerebroprotection

**Figure 8:**
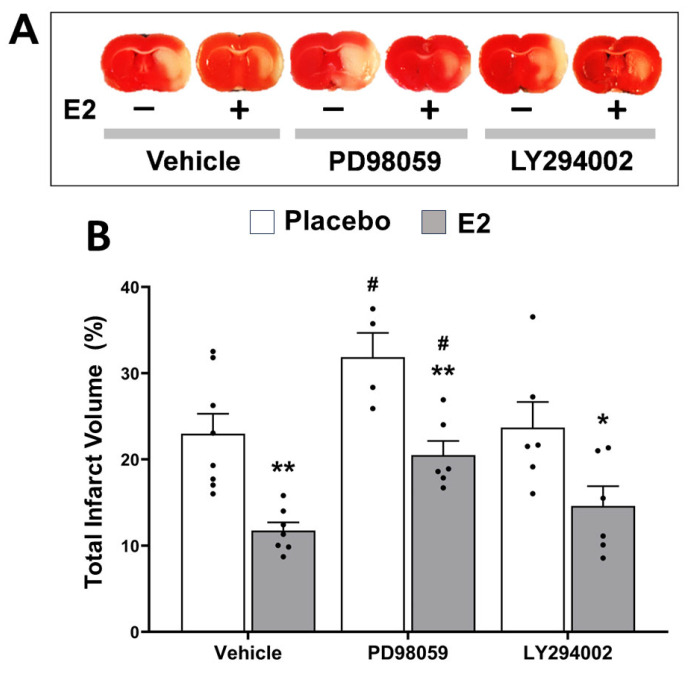
The MEK inhibitor PD98059, but not the PI3K inhibitor LY294002, attenuates estradiol protection. Infarct sizes in brains from placebo- and E2-treated ovariectomized female rats subjected to tMCAO. (**A**) Representative images of the TTC-stained third coronal brain slice from placebo- (−) and E2-treated (+) animals, treated with vehicle, PD98059 or LY294002. (**B**) Summary data showing total infarct volumes, expressed as a percentage of the corresponding non-ischemic hemisphere. Two-way ANOVA for estrogen treatment (E2 vs. placebo, *p* < 0.0001, F_1,31_ = 32.5), and inhibitors treatment (PD98059 and LY294002 vs. vehicle, *p* < 0.01, F_2,31_ = 7.9). Post hoc Sidak’s multiple comparisons tests: significantly different from placebo group (* *p* < 0.05 and ** *p* < 0.01) or from vehicle-treated group (^#^ *p* < 0.05). Data are mean ± SEM of individual data points. E2, 17β-estradiol. tMCAO, transient middle cerebral artery occlusion.

To further gain evidence about the involvement of the MAPK/ERK and/or PI3K/Akt signaling pathways in the E2-induced neuroprotection, animals were also treated with PD98059 (MAPK/ERK pathway inhibitor) and LY294002 (PI3K/Akt pathway inhibitor). In line with results of Experiment I, results of this Experiment II using pathway inhibitors confirmed that E2 treatment significantly reduced the total infarct volume (*p* < 0.001, F_1,31_ = 32.5) vs. placebo treatment. Overall, pathway inhibitors significantly increased infarct volume (*p* < 0.01, F_2,31_ = 7.9) vs. vehicle, thus attenuating E2-induced cerebroprotection (Figure 8A,B). Then, the effects of the two inhibitors were analyzed.

PD98059 treatment significantly enhanced the infarct volume (*p* < 0.05) vs. vehicle treatment. According to the post hoc analysis, PD98059 significantly increased infarct volume when compared to vehicle in both the placebo-treated group (31.9 ± 2.8% in PD98059 vs. 22.96 ± 2.3% in vehicle, *p* < 0.05) and E2-treated group (20.5 ± 1.6% in PD98059 vs. 11.7 ± 1.0% in vehicle, *p* < 0.05), thus abrogating the cerebroprotective effect of E2 (Figure 8A,B).

In contrast, LY294002 treatment did not significantly alter the infarct volume (*p =* 0.9) vs. vehicle treatment. Accordingly, the post hoc analysis showed that E2 significantly reduced the infarct volume when compared to placebo in both the vehicle (11.7 ± 1.0% in E2 vs. 22.96 ± 2.3% in placebo, *p* < 0.01) and LY294002-treated group (14.6 ± 2.3% in E2 vs. 23.7 ± 3.0 in placebo, *p* < 0.05) (Figure 8A,B).

Ultimately, post hoc analysis did not reveal significant variations in infarct volume between the two inhibitors. This was observed in both the placebo (31.9 ±2.8% for PD98059 vs. 23.7 ± 3.0% for LY294002, *p* = 0.08) and the E2-treated groups (20.5 ± 1.6% for PD98059 vs. 14.6 ± 2.3% for LY294002, *p* = 0.2).

## 3. Discussion

Due to the experimental design, our study fits into the still unresolved debate about the risk–benefit balance of HRT as a cerebroprotective approach in stroke. While some clinical studies have demonstrated that estrogen decreases the risk of stroke and the occurrence of cognitive decline after stroke, others suggest that estrogen replacement therapy cannot reduce the risk of mortality and recurrence of stroke in post-menopausal women (see [29] for review). Overall, our results demonstrate that exogenously restored physiological levels of E2 in ovariectomy-induced post-menopausal rats reduces the extent of ischemic stroke-induced brain damage. Such an effect is due to its inhibitory action on cellular apoptosis as well as on the ischemia-reperfusion-induced activation of the MAPK/ERK signaling pathway, and on the PI3K-independent Akt activation. Therefore, our results lend support to the use of HRT as a cerebroprotective strategy against acute ischemic stroke in post-menopausal women. However, as reviewed by Mehta et al. [30], the recommendation is to use HRT by following an individualized approach when treating symptomatic menopausal women with periodic reevaluation that includes the appropriate HRT type, dose, formulation, and route of administration to meet treatment goals for the duration needed.

The first point to consider is the ability of E2 to protect the brain against an episode of ischemia-reperfusion injury in our experimental paradigm. The current results demonstrate that E2 greatly exerts such a cerebroprotective effect in terms of reduction in infarct volume, although it does not reflect an improvement of the neurofunctional score. This is in accordance with previous reports on female animals [31,32]. As for the sex, we have previously reported that E2 also has a neuroprotective effect in male rats [18,19,20]. However, a direct comparison cannot be made as those studies followed the acute treatment approach instead of the preventive approach used in this study. In any case, both the extent of the brain damage and the ability of E2 to reduce it do not seem strikingly different among male and female rats. In males, E2 decreased infarct volume from 27% to 18% [18], and from 25% to 11% [20]. In the current study with female rats, E2 decreased infarct volume from 22% to 12%. Second, assessment of various outcomes was conducted at the 24 h mark following ischemia-reperfusion, a clinically significant timepoint. Evaluating final infarct volume through diffusion-weighted imaging at 24 h has been shown to capture the impact of reperfusion therapies on infarct expansion and to forecast functional outcomes similarly to imaging in the chronic phase (90 days post stroke) [33,34,35]. Conversely, as indicated by the National Institutes of Health Stroke Scale (NIHSS), the 24 h timepoint post symptom onset is pivotal for appraising the clinical state when analyzing the effects of a specific treatment during the stroke’s acute phase. This is corroborated by neurological examinations at 24 h, which have also demonstrated the ability to predict functional outcomes at 1 month [36] and 90 days [37] after the stroke. However, our study has a limitation, which concerns the inclusion of only a 24-h timepoint. This limitation arises from the fact that numerous pathophysiological processes occur during the sub-acute and long-term phases of ischemia. Future research endeavors should encompass long-term assessments and a broader array of functional tests to bolster the validity of our findings. This approach will help illustrate whether the reduction in infarct size may translate into improved functionality over time.

The cellular actions of E2 are related with crosstalk between E2 and growth factor signaling. Activation of the ERK1/2 cascade can be induced by several in vivo and in vitro stimuli, and it is involved in proliferation, cell death, and inflammation [21,38,39]. The role of the MAPK/ERK1/2 pathway in cerebral ischemia is not clear because both beneficial and detrimental events have been reported to be linked to its activation. Several studies show that MAPK/ERK pathway activation can be neuroprotective by suppressing oxidative stress [40] and neuronal apoptosis [41], thus improving stroke outcome. In contrast, other studies indicate that inhibition instead of activation of the pathway protects against apoptosis [21] and inflammation [38,42]. Wakade et al. [43] showed that ERK1/2 phosphorylation significantly increased after MCAO, an event correlated with reduced infarct size. Moreover, ERK inhibitors U0126 and PD184161 have shown to decrease infarct volume after an ischemic insult [24]. A recent study has reported that stimulation of ERK1/2 led to an increase in infarct volume, inflammation, and apoptosis after tMCAO [44], thus indicating that the activation of this pathway is detrimental and that its inhibition is instead neuroprotective. Our results are in close agreement since ischemia-reperfusion significantly increases ERK1/2 phosphorylation/activation in the ischemic hemisphere. Moreover, E2 pretreatment decreases basal phosphorylation of both ERK1 and ERK2 in the non-ischemic hemisphere and prevents ischemia-induced phosphorylation and activation of ERK1/2 in the ischemic hemisphere, which is consistent with findings by Xiao et al. [32]. Such findings show a very different pattern of ERK1/2 activation after the acute E2 treatment when compared to male animals [19,20]. Nonetheless, it is important to note a limitation in this study that could be addressed in future research involving female animals to further extend the insights offered here. Our primary aim was to assess the influence of estradiol at estrus level during tMCAO. To ensure uniformity in serum estradiol levels, our study exclusively involved ovariectomized rats. However, the absence of a non-ovariectomized control group hindered our ability to fully investigate and provide context for findings such as the decrease in pERK1/2 in the non-ischemic hemisphere in E2 versus placebo.

In addition to the MAPK/ERK1/2 pathway, E2 is known to activate PI3K/Akt signaling. Akt plays a central role in this pathway by controlling cell survival and apoptosis [45]. Moreover, it has been reported that while downregulated expression of Akt is related to neurodegeneration, its activation is neuroprotective (for review see [46]). Expression level of pAkt was temporarily increased at early timepoints after ischemia and downregulated after reperfusion at 24 h [47,48], which is consistent with the results of the present study as E2 maintained phosphorylation of Akt after ischemia. Moreover, E2 treatment increased the overall expression of total Akt indicating that E2 may be providing potential benefits by this increase as well. These findings provide evidence implicating the Akt pathway as a cellular mediator of the neuroprotection afforded by E2 pretreatment, at physiological levels, in our model of cerebral ischemia in female animals.

CTMP, an Akt binding protein and endogenous inhibitor, negatively regulates the Akt activity. CTMP binds the carboxyl-terminal regulatory domain of pAkt and suppresses its activity [49]. Our results show that a decrease in pAkt levels at 24 h after the ischemic insult, consistent with the results reported by Won et al. [31], was not accompanied by significant change in the CTMP expression. However, E2 induces CTMP upregulation in the non-ischemic hemisphere that could be triggered by the large increase in pAkt in that hemisphere. Therefore, E2 neuroprotection in our ischemia model is not mediated, most likely, by inhibition of CTMP expression.

Akt is known to phosphorylate BAD at the Ser136 residue, thus triggering its binding to the cytoplasmic retention factor 14-3-3, which ultimately leads to a decrease in apoptotic activity [46]. Our data showing that ischemia-reperfusion induces a decrease in the pBAD level in the ipsilateral hemisphere, and that E2 both prevents the injury-induced decline of pBAD and induces an increase in the pBAD levels in the non-ischemic hemisphere, are consistent with the Akt ability to phosphorylate BAD. This agrees with previous studies showing similar findings [31,50].

Administration of the ERK inhibitor, PD98059, after ischemia-reperfusion exacerbates ischemic damage and reverses the neuroprotection induced by E2. These findings suggest the potential modulation of MAPK/ERK signaling by E2, contributing to the protection of brain cells following ischemia/reperfusion. Furthermore, the ischemic damage exacerbation after inhibition of the MAPK/ERK signaling, in our experimental paradigm, is consistent with observations that ischemia/reperfusion-induced ERK phosphorylation can be both beneficial and detrimental by acting through different receptors or pathways [38]. Alternatively, it is possible that independent influences could overshadow the neuroprotective benefits of E2 pretreatment, resulting in an increase in infarct size due to the inhibition of MAPK/ERK. Additionally, the deprivation of E2 by ovariectomy may somehow magnify the effect of MAPK/ERK signaling inhibition. Even though we know that MEK inhibitor PD098059 reduces transcription of the enzyme aromatase cytochrome p450 [51], which participates in the synthesis of brain E2 from endogenous cholesterol [52], our animals are ovariectomized and do not produce endogenous circulating estrogens. Therefore, the action of circulating E2 in our paradigm can only be due to the administration of exogenous E2, which is the focus of our study.

Our findings acknowledge that E2 pretreatment can maintain Akt signaling after ischemia/reperfusion in female animals, in agreement with other investigators [31,32]. Our results extend these findings by revealing that E2 acts through Akt signaling in a PI3K-independent manner to promote survival. Administration of the PI3K inhibitor, LY294002, does not block the ability of E2 to promote the post-ischemic brain cell survival. This finding documents a novel pathway for E2-induced Akt signaling, in the absence of PI3K activity, to preserve ischemic neurons. Although PI3K is the main pathway of Akt activation, other kinases have been shown to activate Akt directly and these can function even when PI3K activity is inhibited [53]. Another possibility we cannot rule out is that at the concentration used in the present study, LY294002 may not inhibit PI3K. However, we used a concentration of LY294002 previously published in similar experimental paradigms that showed it to be effective in blocking PI3K activity and reversing neuroprotection induced by E2 [54,55].

Taken together, these observations indicate that MAPK/ERK1/2 and Akt signaling pathways are critical while PI3K activation is not essential to E2 pretreatment-mediated cerebroprotection after ischemic stroke in a model of ovariectomy-induced menopause in female rats. These pathways may have common downstream molecules, which prevent activation of caspase cell death cascade, to promote neuronal survival in the face of ischemic insults. The outcomes presented in this study should be interpreted within a broader framework, where it is highly likely that other mechanisms are also at play. Thus, our results pave the way to a better understanding of the mechanisms and the influence of E2 in sex-specific responses, which is needed for the design of successful HRTs.

## 4. Materials and Methods

### 4.1. Animals

A total of 131 female Wistar rats weighing 275–325 g (Charles River, Barcelona, Spain), at the time of ischemic insult, were housed under standard environmental conditions and fed an isoflavone-free chow (TD96155 diet, Harlan Interfauna Ibérica, Sant Feliu de Codines, Barcelona, Spain) with water ad libitum.

### 4.2. Experimental Groups

Ovariectomized female rats were distributed randomly among placebo, E2, and the pharmacological treatments with signaling pathway inhibitors. Experiment I included two groups: (1) placebo pellet and (2) E2 pellet. Experiment II included six groups: (1) placebo pellet + DMSO vehicle, (2) placebo pellet + PD98059, (3) placebo pellet + LY294002, (4) E2 pellet + DMSO vehicle, (5) E2 pellet + PD98059, and (6) E2 pellet + LY294002. Seventy-seven out of one hundred thirty-one rats were excluded from quantitative assessment of stroke outcomes and biomolecular determinations in the study because they fulfilled one of the exclusion criteria: (1) no ischemia (CP reduction < 50% from baseline); (2) no reperfusion (CP did not reach the pre-ischemia baseline after filament withdrawal); (3) no TTC-detectable brain infarction (absence of any pale zone) despite a right ischemia-reperfusion pattern; and (4) death before the 24-h time limit (perioperative mortality) (Table 1).

### 4.3. Ovariectomy and Estradiol Pellet Implantation

All female rats were ovariectomized under anesthesia (face mask, 3.5% sevoflurane in 70% N_2_O plus 30% O_2_) and analgesia (buprenorphine, 0.05 mg/kg, sc). Seven days later, pellets containing 17β-estradiol (0.05 mg/pellet, 21-day sustained release; Innovative Research of America, Inc.; Sarasota, FL, USA) designed to maintain a constant serum level of 30–50 pg/mL or placebo (control, matrix made with cholesterol, cellulose, lactose, phosphates and stearates, Innovative Research of America) were inserted subcutaneously beneath the dorsal surface of the neck. Pellets remained in place from 7 days before ischemia until euthanization. This protocol provides a serum E2 concentration that aligns with estrus levels (30–50 pg/mL), which closely corresponds to values reported in previous studies [56,57].

### 4.4. Transient Focal Cerebral Ischemia

Animals were anesthetized by intraperitoneal injection of 5 mg/kg diazepam, 100 mg/kg ketamine, and 0.3 mg/kg atropine. Anesthesia was maintained with 0.5–1% sevoflurane in 70% N_2_O plus 30% O_2_. Transient right middle cerebral artery occlusion (tMCAO) was performed by following the intraluminal suture procedure as originally described [58] and adapted to our experimental setup [59]. This includes continuous monitoring of cerebrocortical laser-Doppler flow (cortical perfusion, CP), arterial blood pressure (ABP) and core temperature (T), and discontinuous measurement of pH, PaO_2_, PaCO_2_, and glucose at pre-ischemia (basal), ischemia, and reperfusion stages. MCAO was maintained for 60 min, after which reperfusion was monitored for 30 min. Twenty-four hours after the ischemic insult, the animals were subjected to neurofunctional evaluation and euthanized by intracardiac injection of KCl (200 mg/kg) under anesthesia to obtain the brain according to specific requirements for each determination.

### 4.5. PD98059 and LY294002 Intracerebroventricular Administration

Anesthetized animals were treated with intracerebroventricular (icv) administration of the selective MAPK kinase (MEK) inhibitor PD98059 (3 µg, Calbiochem, La Jolla, CA, USA), the potent and specific PI3K inhibitor LY294002 (30 µg, Promega; Madison, WI) or vehicle (10 µL of 50% DMSO). The concentrations of the inhibitors used in this study were based on the previously published experimental paradigms of cerebral ischemia, which showed to be effective in blocking MAPK and PI3K activities. In addition, both inhibitors reversed neuroprotection induced by E2 in these studies [54,55,60]. Icv injections of up to 75% DMSO have no obvious harmful effects [60,61]. To avoid low penetration of these drugs across the blood–brain barrier [62], treatments were applied through a brain infusion kit (Alzet, Durect Corp., Cupertino, CA, USA), stereotaxically targeting the right lateral ventricle to a position defined by the following coordinates (according to the atlas of Paxinos and Watson): 1 mm posterior to bregma, 1.5 mm lateral to bregma, and 4.5 mm below the skull surface [63]. Manually controlled infusion was carried out at a flow rate of 10 µL/min by means of a Hamilton syringe connected to the brain infusion cannula through a vinyl catheter tube. Injection was carried out 30 min after filament withdrawal, i.e., at 90 min from the onset of ischemia.

### 4.6. Neurofunctional Evaluation and Infarct Volume Measurement

Just before euthanasia, a neurofunctional evaluation was performed based upon four criteria [59]: (a) spontaneous activity (moving/exploring = 0, moving without exploration = 1, and not moving or only when pulled by the tail = 2); (b) circling to the left (none = 0, when elevated by the tail and pushed or pulled = 1, spontaneously = 2, and circling without displacement (spinning top) = 3); (c) parachute reflex by protective abduction of forelimbs (symmetrical = 0, asymmetrical = 1, and non-ischemic forelimb retracted =2); and (d) resistance to left forepaw stretching (stretching not allowed = 0, stretching allowed = 1, and no resistance = 2). The total score could range from 0 (no neurological deficits) to 9 (highest neurological deficits).

Brain infarct volume was determined using the 2,3,5-triphenyltetrazolium chloride (TTC) vital staining method [64] followed by morphometric analysis [59]. In short, the rats were euthanized, and the brain was sliced in seven 2 mm thick coronal sections, which were immersed in a 2% solution of TTC in saline solution at 37 °C for 15 min, fixed in 10% phosphate-buffered formalin (pH 7.4) overnight, and digitally photographed for image analysis. Infarct area in the slices and the total infarct volume were calculated with edema correction, separately for cortical and subcortical regions, obtained as absolute values (mm^3^) and expressed as a percentage of the corresponding non-ischemic region. Experimenters performing neurofunctional score and infarct volume assessments were blinded to the treatments.

### 4.7. Serum Estradiol Assay

Tubes containing whole blood were placed on ice (10 min) and centrifuged at 300× *g* for 5 min. Serum was collected and stored (−20 °C) until analyzed. Serum hormone levels were measured using an enzyme immunoassay using the DRG estradiol sensitive ELISA kit (DRG Instruments GmbH; Marburg, Germany). All assays were performed in duplicate, and the mean value reported. The range of the assay is between 0–200 pg/mL.

### 4.8. Detection of DNA Cleavage

Brains were frozen and cut into 18 μm sections in the coronal plane (0.2 to −1.8 mm from bregma). TUNEL labeling (three brain sections per animal) was performed using an in situ cell death detection kit TMR red, as per the manufacturer’s instructions (Roche Molecular Biochemicals, Mannheim, Germany). The kit contains terminal deoxynucleotidyl transferase (TdT) that catalyzes polymerization of TMR red dUTP to free 3′-OH DNA ends in a template independent manner. TUNEL-positive cells are identified directly by fluorescence of incorporated dUTP. Brain sections were viewed under a LEICA DM 4500B (Leica Microsystems, Barcelona, Spain) fluorescence microscope. Images were acquired with a LEICA DFC 300 FX camera with LEICA application suite V4. For quantification of TUNEL reactivity, three fields of the cortical region of the ischemic hemisphere were selected (see Figure 2 and its legend) and the number of TUNEL-positive cells for each field was counted using ImageJ-win32 (NIH, Bethesda, MD, USA). The number of TUNEL-positive cells, from each of the three sections, was averaged to provide a single value for each animal, expressed as a percentage of the corresponding DAPI-stained nuclei and averaged for each ischemic cortex. The individual conducting the experiments was blinded to the identity of the treatment group during both image acquisition and quantification.

### 4.9. Caspase Activity Assay

Caspase activity assay was performed on frozen brain sections, from the same samples used in the TUNEL assay, by using the APO LOGIX™ carboxyfluorescein (FAM) caspase detection kit (Cell Technology, Minneapolis, MN, USA). FAM-DEVD-FMK is a carboxy-fluorescein-tagged analogue of zDEVD-fluoromethyl ketone (FMK), a broad-spectrum cysteine protease inhibitor that enters cells and irreversibly binds to catalytically active caspase-3 [19,60,65,66]. Briefly, brain sections (three per animal) were labeled with 5 μM FAM-DEVD-FMK (1 h, 37 °C), washed three times with wash buffer, fixed, and cover slipped with ProLong (Molecular Probes, Eugene, OR, USA). To quantify caspase-3 activity, the fluorescence within three selected fields of the ischemic cortex (as shown in Figure 3 and its legend) was quantified for each individual section. Image viewing, acquisition, and analysis were also conducted as described above. The fluorescence from each of the three sections was averaged to provide a single value for each animal, expressed as a percentage of the field area and averaged for each ischemic cortex.

### 4.10. Western Blot Analysis

Western blot analysis was performed for quantitation of protein abundance in the non-ischemic versus ischemic hemisphere. A 2 mm thick brain coronal section (0.2 to −1.8 mm from bregma) was obtained, and ischemic and non-ischemic hemispheres were separated. Tissue was homogenized in lysis buffer (ProteoJetTM Mammalian Cell Lysis Reagent, Fermentas, Burlington, ON, Canada) containing protease and phosphatase inhibitor cocktails (1%, Sigma-Aldrich). Protein concentration was determined using BCA protein assay kit (Pierce, Rockford, IL, USA). Aliquots of protein (40 μg) were dissolved in NuPAGE LDS sample buffer (Invitrogen, Carlsbad, CA, USA) under reducing conditions, loaded on 4–12% Bis-Tris gels (Invitrogen), subjected to SDS-PAGE and electrotransferred to 0.2 μm nitrocellulose membranes for immunolabeling using the following primary antibodies: (1) anti-cleaved caspase-3 (Asp175), rabbit polyclonal antibody that detects endogenous levels of the large fragment (17/19 KDa) of activated caspase-3 (#9661;1:500; Cell Signaling Technology, Inc., Beverly, MA, USA); (2) anti-phospho MAPK (pERK1/2) rabbit monoclonal antibody, which recognizes ERK1 and ERK2 that are phosphorylated on both a Thr202 and a Tyr204 residue (D13.14.4E; #4370;1:2000; Cell Signaling Technology); (3) anti-MAPK1/2 (ERK1/2) rabbit polyclonal antibody (#06-182; 1:5000; Millipore, Temecula, CA, USA); (4) anti-phospho BAD (pBAD) rabbit monoclonal antibody, which detects endogenous levels of BAD only when phosphorylated at Ser136 (D25H8; #4366;1:500; Cell Signaling Technology); (5) anti-Bad rabbit polyclonal antibody (#9292; 1:500; Cell Signaling Technology); (6) anti-phospho Akt (pAkt) mouse monoclonal antibody, which recognizes Akt only when phosphorylated at Ser473 (193H12; #4058; 1:000; Cell Signaling Technology); (7) anti-Akt (Akt, pan) rabbit monoclonal antibody (C67E7; #4691; 1:1000, Cell Signaling Technology); (8) anti-CTMP, rabbit polyclonal antibody, which detects endogenous levels of total CTMP protein (#4612; 1:500; Cell Signaling Technology); and (9) anti-β-actin mouse monoclonal antibody (Clone AC-15, #A5441; 1:10000; Sigma, Saint Louis, MI, USA). Secondary antibodies for Western blot were horseradish peroxidase (HRP)-conjugated goat anti-rabbit IgG (1:5000, Bio-Rad, Hercules, CA, USA) for rabbit antibodies, or goat anti-mouse IgG (1:2000, Bio-rad) for mouse antibodies.

After reaction, membranes were treated with enhanced chemiluminescence reagents (ECL, Amersham Life Science, Buckinghamshire, UK) and imaged using ChemiDoc XRS imaging system (Bio-Rad). Membranes were re-probed with anti-β-actin antibody as a loading control.

To quantitate protein abundance, bands on Western blots were analyzed using NIH IMAGE 1.61 software. Band densities for pERK1, pERK2, p-Akt, and p-Bad were corrected for variations in loading and normalized to the corresponding band densities for total ERK1, total ERK2, total Akt, and total Bad, respectively. Band densities for caspase-3 and CTMP were corrected for variations in loading and normalized to the corresponding band densities for β-actin.

### 4.11. Statistical Analysis

The results of quantitative continuous variables were expressed as mean ± SEM. Categorical ordinal neurological scores were expressed as the median (Q1, Q3). Data analysis was performed using GraphPad Prims version 9.00 (GraphPad Software Inc. San Diego, CA, USA). Statistical comparisons were made between groups using Mann–Whitney test (for neurological scores), unpaired Student-t test (for serum E2, total infarct volume, TUNEL, and caspase-3 activity), two-way ANOVA followed by Sidak’s multiple comparisons post hoc test (for regional infarct volume and total infarct volume in experiments with a signaling pathway inhibitor, and for immunoblots). Differences were considered significant at *p* < 0.05.

## Figures and Tables

**Table 1:** Animals excluded from the study according to the established criteria.

		tMCAO	1	2	3	4	Final n
**Placebo**	Control (17)	No treatment	0	0	2	6	9
	Treatment (38)	**Vehicle** (17)	2	1	3	3	8
		PD 98059 (9)	1	1	1	2	4
		LY 244002 (12)	2	1	1	2	6
**17-β-Estradiol**	Control (28)	No treatment	0	3	10	7	8
	Treatment (48)	**Vehicle** (26)	0	2	9	8	7
		PD 98059 (12)	0	1	2	3	6
		LY 244002 (10)	1	0	1	2	6

(1) No ischemia, (2) No reperfusion, (3) No infarction, (4) Death.

## Data Availability

The datasets of this study are available from the corresponding author upon reasonable request.
